# The kinesin KIF1C transports APC-dependent mRNAs to cell protrusions

**DOI:** 10.1261/rna.078576.120

**Published:** 2021-12

**Authors:** Xavier Pichon, Konstadinos Moissoglu, Emeline Coleno, Tianhong Wang, Arthur Imbert, Marie-Cécile Robert, Marion Peter, Racha Chouaib, Thomas Walter, Florian Mueller, Kazem Zibara, Edouard Bertrand, Stavroula Mili

**Affiliations:** 1 Institut de Génétique Moléculaire de Montpellier, University of Montpellier, CNRS, 34293 Montpellier, France; 2 Equipe labélisée Ligue Nationale Contre le Cancer, University of Montpellier, CNRS, 34000 Montpellier, France; 3 Laboratory of Cellular and Molecular Biology, Center for Cancer Research, National Cancer Institute, NIH, Bethesda, Maryland 20814, USA; 4 Institut de Génétique Humaine, University of Montpellier, CNRS, 34396 Montpellier, France; 5 MINES ParisTech, PSL-Research University, CBIO-Centre for Computational Biology, 77300 Fontainebleau, France; 6 Institut Curie, 75248 Paris Cedex, France; 7 INSERM, U900, 75248 Paris Cedex, France; 8 Biology Department, Faculty of Sciences-I, Lebanese University, Beirut, Lebanon; 9 Unité Imagerie et Modélisation, Institut Pasteur and CNRS UMR 3691, 75015 Paris, France; 10 C3BI, USR 3756 IP CNRS – Paris, France; 11 ER045, PRASE, DSST, Lebanese University, Beirut, Lebanon

**Keywords:** RNA localization, local translation, RNA transport, cytoplasmic protrusions

## Abstract

RNA localization and local translation are important for numerous cellular functions. In mammals, a class of mRNAs localize to cytoplasmic protrusions in an APC-dependent manner, with roles during cell migration. Here, we investigated this localization mechanism. We found that the KIF1C motor interacts with APC-dependent mRNAs and is required for their localization. Live cell imaging revealed rapid, active transport of single mRNAs over long distances that requires both microtubules and KIF1C. Two-color imaging directly revealed single mRNAs transported by single KIF1C motors, with the 3′UTR being sufficient to trigger KIF1C-dependent RNA transport and localization. Moreover, KIF1C remained associated with peripheral, multimeric RNA clusters and was required for their formation. These results reveal a widespread RNA transport pathway in mammalian cells, in which the KIF1C motor has a dual role in transporting RNAs and clustering them within cytoplasmic protrusions. Interestingly, KIF1C also transports its own mRNA, suggesting a possible feedback loop acting at the level of mRNA transport.

## INTRODUCTION

Localization of mRNA to specific subcellular compartments is an important mechanism for the spatio-temporal regulation of gene expression in diverse cell types and organisms (Chin and Lécuyer 2017; Eliscovich and Singer 2017). Subcellular mRNA localization allows localized protein synthesis and this is important for many biological functions such as cell fate determination (Berleth et al. 1988), cell polarization (Condeelis and Singer 2005), cell division (Chouaib et al. 2020; Ryder et al. 2020), cell migration (Katz et al. 2012; Wang et al. 2017; Moissoglu et al. 2020), embryonic patterning (Forrest and Gavis 2003), and synaptic plasticity (Martin and Zukin 2006; Lin and Holt 2007). One of the best characterized examples is the yeast *Ash*1 mRNA that localizes specifically in the bud of the daughter cells and encodes a transcriptional repressor protein involved in suppressing mating-type switching (Paquin and Chartrand 2008). Studies of this and other models revealed that the subcellular localization of mRNA relies on three main mechanisms, acting separately or in combination: random diffusion combined with local entrapment, general transcript degradation coupled to localized protection and directed transport along the cytoskeleton (Medioni et al. 2012; Cody et al. 2013; Bovaird et al. 2018).

Active, motor-driven transport of mRNAs along the cytoskeleton is thus far the most common localization mechanism. It generally involves *cis*-acting elements, also called zipcodes, contained in the 3′UTR sequence of the transcript. This is exemplified by the case of the β-actin mRNA in vertebrates, which accumulates at the leading edge of migrating cells and was among the first localized mRNAs discovered (Singer 1993). This mRNA contains a zipcode sequence recognized by the RNA Binding Protein ZBP1, allowing the transport of β-actin mRNAs in a motor-driven manner along the cytoskeleton (Kislauskis et al. 1993; Oleynikov and Singer 2003; Condeelis and Singer 2005; Liao et al. 2015). Interestingly, transport of β-actin mRNA by ZBP1 involves both microtubules (MTs) and actin filaments (Fusco et al. 2003; Oleynikov and Singer 2003), as well as several motors that display some cell type and compartment specificity. Indeed, MYO5A and KIF5A interact with ZBP1 to transport β-actin mRNAs in dendrites and axons (Ma et al. 2011; Nalavadi et al. 2012), while Myosin IIB (MYH10) and KIF11, which directly binds ZBP1, regulate the transport of β-actin mRNAs in fibroblasts and during cell migration (Latham et al. 2001; Song et al. 2015).

In vertebrate systems, the motors involved in RNA transport have been investigated mostly in neuronal cells. Kinesin-1 (KIF5) was shown to associate with neuronal RNP granules and to be involved in their trafficking (Kanai et al. 2004). Kinesin-1 was also implicated in transport of myelin basic protein (MBP) mRNA in oligodendrocytes (Carson et al. 1997) as well as in *shank1* mRNA transport in rat neurons (Falley et al. 2009). Kinesin-2 (KIF3A/B/KAP3) can transport RNAs in vitro (Baumann et al. 2020), but its in vivo relevance is still unclear. The involvement of additional motors, and the means through which they connect to potential RNA cargoes are still largely unexplored, especially in the case of nonneuronal cell types (Gagnon and Mowry 2011; Xing and Bassell 2013).

Localized RNAs are prevalent in nonneuronal, mesenchymal cells. Apart from β-actin, numerous other RNAs are localized at protrusions of mesenchymal cells and their local translation is important for cell migration (Mili et al. 2008; Mardakheh et al. 2015; Costa et al. 2020; Moissoglu et al. 2020). Localization of these RNAs is carried out through at least two distinct pathways. Specifically, a subset of about a hundred RNAs, which include transcripts encoding signaling and cytoskeleton regulators (such as the Rab GTPase RAB13, the RhoA exchange factor NET1, the collagen receptor DDR2, the motor related proteins TRAK2, DYNLL2, and others), require the APC tumor suppressor protein for localization and have been referred to as APC-dependent (Wang et al. 2017). Other protrusion-enriched RNAs, exemplified by RNAs encoding ribosomal proteins, do not require APC and exhibit distinct regulation (Wang et al. 2017).

Similar to what has been described for other localized RNAs, sequences within the 3′UTR of APC-dependent RNAs are necessary and sufficient for targeting to the cell periphery (Mili et al. 2008). Specifically, interfering with or deleting particular GA-rich regions is sufficient to disrupt peripheral localization and perturb cell movement in various systems (Chrisafis et al. 2020; Costa et al. 2020; Moissoglu et al. 2020). Furthermore, localization to the periphery requires the microtubule cytoskeleton and in particular a subset of stable, detyrosinated microtubules (Wang et al. 2017; Moissoglu et al. 2019). Indeed, at least some APC-dependent RNAs exhibit a colocalization with the plus ends of detyrosinated microtubules (Mili et al. 2008). The peripheral complexes also contain APC, a protein that has the ability to directly bind microtubules via its carboxyl terminus (Munemitsu et al. 1994; Zumbrunn et al. 2001; Jimbo et al. 2002; Barth et al. 2008; Bahmanyar et al. 2009), hence suggesting that APC might mediate the interaction of localized mRNAs with microtubules (Mili et al. 2008; Preitner et al. 2014).

An additional feature integrated with the localization of APC-dependent RNAs is their existence in distinct physical states. In particular, RNAs in internal or peripheral, actively extending cytoplasmic regions exist as single molecules that are undergoing translation. However, at some peripheral areas, single RNAs coalesce in multimeric heterogeneous clusters that are composed of multiple distinct RNA species. Interestingly, these clusters preferentially form at retracting protrusions and contain translationally silent mRNAs (Moissoglu et al. 2019). These data indicate the existence of a dynamic regulatory mechanism during cell migration, which coordinates local mRNA translation with protrusion formation and retraction. However, the exact mechanisms and molecular players involved in transport to the periphery and cluster formation for this group of RNAs are still unclear.

In this study, we focused on the kinesin KIF1C, which we recently showed to accumulate and colocalize with its own mRNA in cytoplasmic protrusions (Chouaib et al. 2020). We show here that KIF1C associates with additional protrusion-localized RNAs belonging to the APC-dependent group. We describe a specific mRNA transport mechanism by which the KIF1C kinesin motor binds APC-dependent mRNAs, including its own, actively transports them to cell protrusions in a 3′UTR dependent manner and additionally participates in promoting and/or maintaining their peripheral clusters.

## RESULTS

### Identification of a specific mRNA subset associating with the KIF1C motor in human cells

**FIGURE 1.**
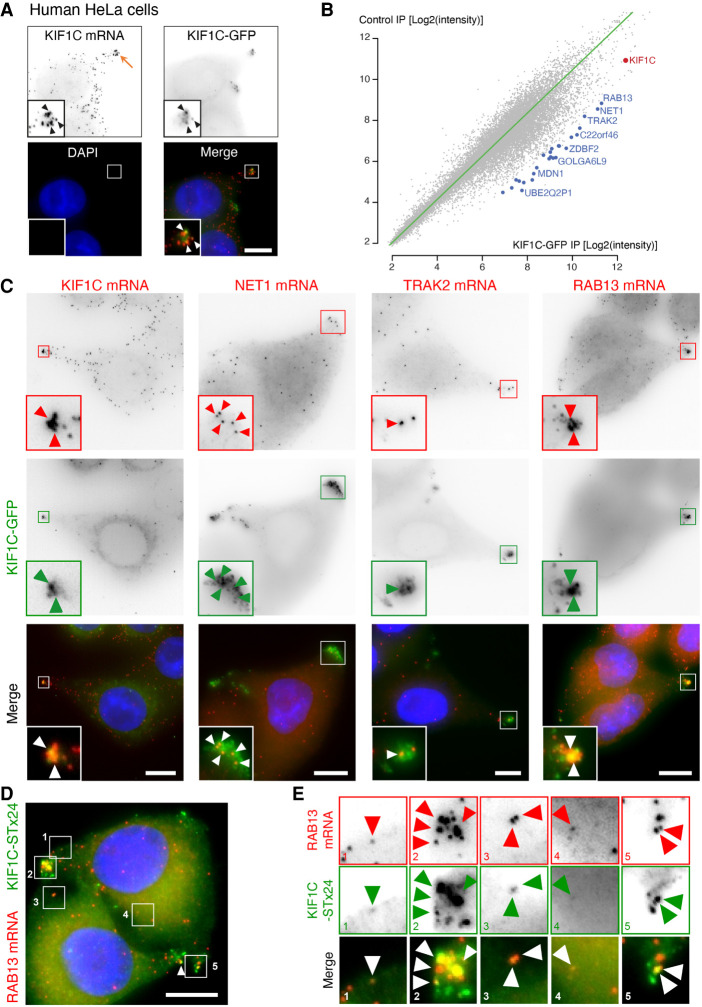
Identification of mRNAs associated with the KIF1C motor. (*A*) The KIF1C kinesin colocalizes with its mRNA in protrusions of HeLa cells. Images are micrographs of a H9 FlipIn HeLa cell line stably expressing a KIF1C-GFP cDNA. (*Top left*) *KIF1C* mRNA detected by smFISH with probes against the endogenous mRNA; (*top right*) KIF1C-GFP protein; (*bottom left*) DNA stained with DAPI; (*bottom right*) merge of the two signals with the KIF1C-GFP protein in green and *KIF1C* mRNA in red. Orange arrow: a cell protrusion. Blue: DNA stained with DAPI. Scale bar: 10 microns. *Insets* represent zooms of the boxed areas in the merge panel. White and black arrowheads indicate the colocalization of *KIF1C* mRNA with KIF1C-GFP protein. (*B*) Transcripts associating with the KIF1C-GFP protein. The graph depicts the microarray signal intensity of RNAs detected in a KIF1C-GFP pull-down (*x*-axis), versus the control IP (*y*-axis). Each dot represents an mRNA. Red dot: *KIF1C* mRNA; blue dot: mRNAs enriched in the KIF1C-GFP IP. *N* = 2. (*C*) Colocalization of KIF1C-GFP with *KIF1C*, *NET1*, *TRAK2*, and *RAB13* mRNAs. Images are micrographs of HeLa H9 Flip-In cells stably expressing a KIF1C-GFP cDNA, labeled by smiFISH with probes against the indicated mRNAs. (*Top*) Cy3 fluorescent signals corresponding to endogenous *KIF1C*, *NET1*, *TRAK2*, and *RAB13* mRNAs. (*Middle*) KIF1C-GFP signal. (*Lower*) Merge with the Cy3 signal in red and GFP signal in green. Blue: DNA stained with DAPI. Scale bar: 10 microns. Arrowheads indicate accumulation of single mRNA molecules at cell protrusions. (*D*) Single-molecule colocalization of KIF1C-ST-x24 with *RAB13* mRNAs. Images are micrographs of Hela cells stably expressing KIF1C-STx24 and scFv-GFP. Red: Cy3 fluorescent signals corresponding to *RAB13* mRNAs labeled by smiFISH with probes against endogenous *RAB13* mRNA. Green: GFP signal corresponding to single molecules of KIF1C protein. Blue: DNA stained with DAPI. Scale bar: 10 microns. (*E*) *Insets* represent zooms of the numbered areas from panel *D*. Legend as in *D*. Arrowheads indicate molecules of *RAB13* mRNA and KIF1C-STx24 protein. Micrographs show cells representative of the population.

High-throughput mRNA–protein cross-linking approaches previously showed that KIF1C directly binds mRNAs (Baltz et al. 2012; Castello et al. 2012), and we recently showed that KIF1C mRNAs and proteins colocalize together in protrusions of HeLa cells (Fig. 1A; Chouaib et al. 2020), suggesting that the KIF1C kinesin might be somehow involved in the metabolism of protrusion mRNAs. To determine the identity of the mRNAs bound by the KIF1C motor, we used a HeLa cell line stably expressing a KIF1C-GFP fusion from a bacterial artificial chromosome containing all the regulatory sequences of the human KIF1C gene, including its 5′ and 3′UTRs (Poser et al. 2008; Chouaib et al. 2020). We immunoprecipitated (IP) KIF1C-GFP with anti-GFP antibodies or uncoated beads as controls, and identified the coprecipitated RNAs using microarrays (Fig. 1B; Supplemental Table S1). We found that many mRNAs were enriched in the KIF1C-GFP IP as compared to the control IP. To explore in more detail the localization of the mRNAs associated with KIF1C, we performed a small smFISH localization screen in HeLa cells. We tested the 26 most enriched mRNAs in the KIF1C IP (Supplemental Tables S1, S2) and found four that were peripherally enriched. These included the *RAB13* mRNA (5.7-fold enrichment, Supplemental Table S1), along with the *KIF1C* mRNA itself (2.6-fold enrichment) and the *NET1* and *TRAK2* mRNAs (5.7- and 4.9-fold enrichment, respectively). We had previously reported that these transcripts localize to protrusions of mouse cells in an APC-dependent manner (Wang et al. 2017), and we thus focused on them. Visual examination of the images revealed that *KIF1C*, *NET1*, *TRAK2*, and *RAB13* mRNAs clearly localized also to protrusions of HeLa cells (Supplemental Fig. S1A). To confirm the link between APC-dependent mRNA localization and binding to KIF1C protein, we performed a correlation analysis of the two metrics (Supplemental Fig. S1B; Supplemental Table S3). This indicated that APC-dependent mRNAs indeed preferentially associate with KIF1C protein, while mRNAs coding for ribosomal proteins, which often localize to protrusions independently of APC (Wang et al. 2017), do not. The IP/microarray data thus show a physical link between KIF1C and mRNAs that localize to protrusions in an APC-dependent manner.

Next, we tested whether these mRNAs colocalize with the KIF1C protein in vivo. To this end, we performed smFISH experiments in a HeLa cell line stably expressing a KIF1C-GFP mRNA from a cDNA and found that indeed, *KIF1C*, *NET1*, *TRAK2*, and *RAB13* mRNAs colocalized with the KIF1C-GFP protein in cytoplasmic protrusions (Fig. 1C). In order to show that this colocalization reflected a molecular interaction, we performed single-molecule imaging using the SunTag system (Tanenbaum et al. 2014). To this end, we generated a stable HeLa cell line expressing KIF1C-fused to 24 repeats of the GCN4 epitope (KIF1C-SunTag_x24_), together with the single-chain variable fragment fused to sfGFP (scFv-sfGFP). This system enables the detection of single molecules of the KIF1C protein (Fig. 1D; Tanenbaum et al., 2014). We thus combined detection of single KIF1C-SunTag_x24_ proteins with single mRNA detection by smFISH, using probes against *RAB13* and *NET1* mRNA, or *CRM1* and *RBP1* mRNAs as controls (Fig. 1D,E; Supplemental Fig. S1C–E). KIF1C-SunTag_x24_ proteins were found to colocalize with *RAB13* and *NET1* mRNAs at protrusions as expected, while the control mRNAs did not. In addition, we also observed colocalization of KIF1C-SunTag_x24_ with both *RAB13* and *NET1* mRNAs at the single-molecule level at more internal locations in the cytoplasm (Fig. 1E, panels 1, 3,4; Supplemental Fig. S1D). This confirmed the interaction of single molecules of KIF1C protein with single molecules of *RAB13* mRNAs. Taken together, these data raise the possibility that the kinesin KIF1C might be part of a mechanism that localizes APC-dependent mRNAs to cytoplasmic protrusions.

### KIF1C associates with APC and is required for the localization of APC-dependent mRNAs to cytoplasmic protrusions in human and mouse cells

**FIGURE 2.**
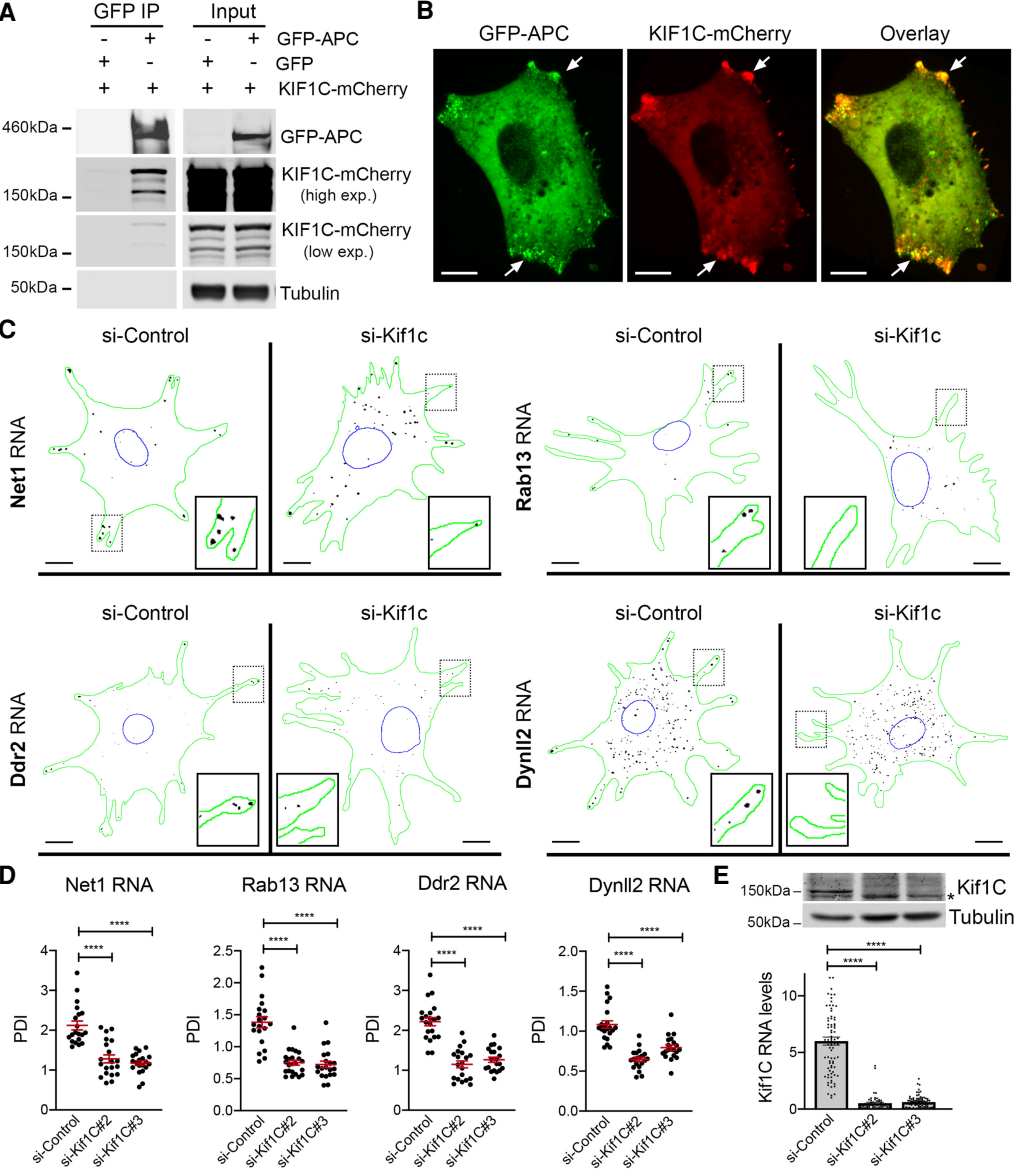
KIF1C associates with APC and is required for the localization of APC-dependent mRNAs to cytoplasmic protrusions. (*A*) Coimmunoprecipitation of KIF1C with APC. GFP or GFP-APC were immunoprecipitated from cells also expressing KIF1C-mCherry and analyzed by western blot to detect the indicated proteins. Results are representative of three independent experiments. (*B*) Colocalization of GFP-APC and KIF1C-mCherry at cytoplasmic protrusions (arrows). Images are representative of multiple cells observed in two independent experiments. Scale bar: 10 microns. (*C*) Depletion of Kif1c prevents mRNA accumulation in protrusions. Images are micrographs of NIH/3T3 cells labeled by smFISH with probes against *Net1*, *Rab13*, *Ddr2*, and *Dynll2* mRNAs, following treatment with siRNAs against Kif1C (panels si-Kif1c) or a control sequence (panel si-Control). Scale bars: 10 microns. Green: outline of the cells; blue: outline of the nuclei; black: smFISH signals. *Insets* represent magnifications of the boxed areas. (*D*) Quantification of mRNA localization of cells described in *C*. Graphs represent the intracellular distribution of the indicated mRNAs as measured by PDI index, with and without treatment of cells with the indicated siRNAs. Red bars represent the mean and 95% confidence interval. Points indicate individual cells observed in two independent experiments. (*E*) Detection of Kif1C protein (*upper* panels) or Kif1C RNA levels (*lower* graph) from cells treated with the indicated siRNAs. Stars in *D* and *E* are *P*-values: (****) *P* < 0.0001, (***) *P* < 0.001, estimated by analysis of variance with Bonferroni's multiple comparisons test.

To further support the connection between KIF1C and APC-dependent RNAs we tested whether KIF1C and APC interact in cells. Indeed, immunoprecipitation of GFP-APC revealed a specific association with KIF1C-mCherry (Fig. 2A). We additionally imaged the two fluorescent proteins in live cells and detected a colocalization between GFP-APC and KIF1C-mCherry in peripheral clusters (Fig. 2B). Therefore, KIF1C exhibits a specific association with both APC protein and peripherally localized APC-dependent RNAs.

To test whether the localization of APC-dependent mRNAs in protrusions depended on the KIF1C protein, we depleted KIF1C by multiple siRNAs in HeLa or MDA-MB-231 cells, and performed smFISH experiments using probes against *RAB13* or *NET1* mRNAs. Intracellular distributions of mRNAs were quantitatively assessed by calculating a Peripheral Distribution Index (PDI), a metric that distinguishes diffusely distributed from peripherally localized RNAs, by measuring the distance of the RNA signal relative to the centroid of the nucleus (Wang et al. 2017; Stueland et al. 2019). For each cell, the RNA distribution is normalized to a hypothetical uniform distribution such that a PDI value of 1 indicates a uniform, diffuse signal, while values smaller or greater than 1 indicate a perinuclear or peripheral localization, respectively. Remarkably, both *RAB13* and *NET1* mRNAs became less localized when KIF1C expression was reduced with siRNAs (Supplemental Fig. S2A–D), demonstrating that the KIF1C kinesin was required for mRNA localization in human cells.

Next, we moved to a mouse system, NIH/3T3 cells, where the localization of mRNAs in protrusions has been extensively studied (Chicurel et al. 1998; Mili et al. 2008; Wang et al. 2017; Moissoglu et al. 2019). To test whether the KIF1C protein has a general role in localizing mRNAs at cell protrusions, we assessed the localization of a series of APC-dependent and APC-independent mRNAs by smFISH, following depletion of KIF1C expression with two different siRNAs. As shown in Figure 2C–E and Supplemental Figure S2E,F, several APC-dependent RNAs, including *Net1, Rab13, Ddr2, DynII2*, and *Cyb5r3,* exhibited a protrusion localization pattern that was lost following KIF1C depletion. Indeed, KIF1C loss led to RNA distributions that were mostly diffuse (PDI values centering around 1; Fig. 2B; Supplemental Fig. S2F), indicating that the KIF1C motor has an important contribution toward directing peripheral mRNA localization. Interestingly, the localization of two APC-independent mRNAs, *Rps20* and *Rpl27a*, was not affected (Supplemental Fig. S2G). To ascertain that this effect was not due to altered mRNA expression, we measured their levels following KIF1C depletion (Supplemental Fig. S2H). This analysis showed no changes in the overall abundance of APC-dependent mRNAs, except for the depleted *KIF1C* mRNA (Supplemental Fig. S2H). Therefore, we conclude that KIF1C is required for the localization of APC-dependent mRNAs to cell protrusions, in various human and mouse cells.

### KIF1C is required for active transport of APC-dependent mRNAs on microtubules

**FIGURE 3.**
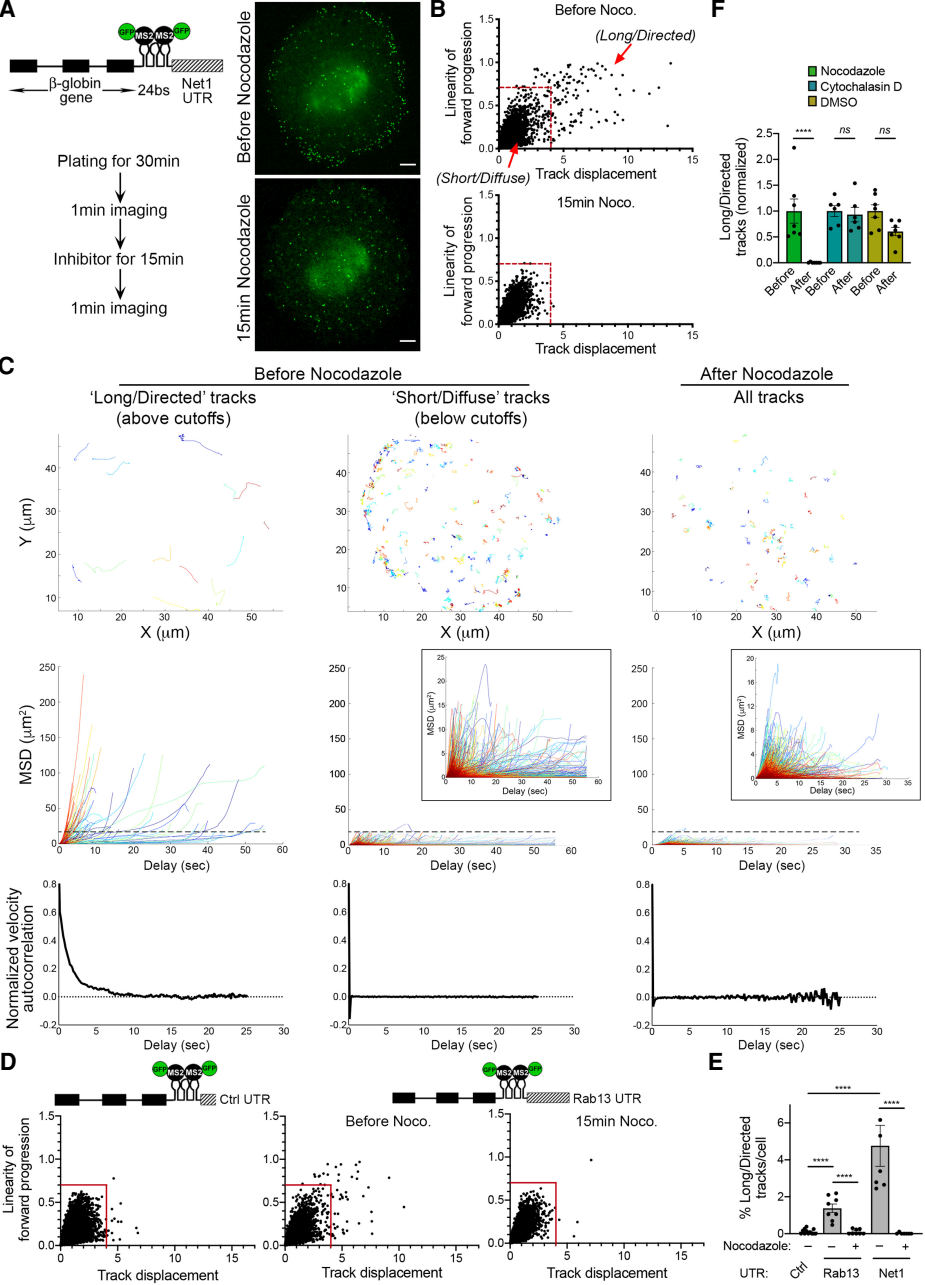
Reporter mRNAs containing *Net1* and *Rab13* 3′UTRs display long, directed microtubule-dependent displacements. (*A*) Schematic of the mRNA reporter construct containing the β-globin coding sequence followed by 24xMS2 binding sites and the mouse *Net1* 3′UTR (β24bs/Net1). Images are snapshots of live NIH/3T3 before or after nocodazole treatment, following the experimental scheme detailed on the *left*. High speed imaging was performed over 1 min to track individual RNA movements. See Supplemental Movies S1, S2 for time lapse imaging. The cells stably expressed the β24bs/Net1 reporter mRNA and MCP-GFP (Green). Scale bar: 5 microns. (*B*) The graphs plot the displacement over the linearity of forward progression (defined as the mean straight line speed divided by the mean speed) of single RNA tracks from cells treated as described in *A*. Red lines indicate the thresholds used to separate “long/directed” from “short/diffuse” tracks. *N* = 6–7 cells. (*C*) Analysis of “long/directed” or “short/diffuse” tracks, from untreated cells, or of all tracks from nocodazole-treated cells, from *B*. Individual raw tracks from a representative cell are displayed in the *upper* panels. Mean square displacement (MSD) (*middle* panels) and velocity autocorrelation (*bottom* panels) of tracks are shown. *N* = 180 (long/directed); 4427 (short/diffuse); 3795 (after nocodazole) tracks from seven cells. Note that “long/directed” tracks exhibit higher MSD (>15 µm^2^, dashed black line), than “short/diffuse” or nocodazole-treated tracks, as well as positive velocity autocorrelation. *Insets* of MSD plots present zooms of *y*-axis scale. (*D*) Single RNA molecules of the β24bs/Control 3′UTR (Ctrl) or the β24bs/Rab13 3′UTR reporters were tracked over 1 min period in cells treated or not with nocodazole. Graphs plot the displacements of individual tracks (*x*-axis) over the linearity of their forward progression (*y*-axis). Red lines indicate the thresholds used to filter tracks of molecules undergoing directed movement. *N* = 8–12 cells. (*E*) The graph depicts the percentage of long/directed tracks of the indicated reporters per cell following treatment with nocodazole. Stars represent *P*-values: (****) *P* < 0.0001, estimated using one-way analysis of variance with Sidak's multiple comparisons test. Error bars: standard error of the mean. (*F*) The bar plot depicts the percentage of long/directed tracks per cell before and after treatment with the indicated compounds. Average values of respective “Before” values were set to 1. *N* = 6–7. Stars represent *P*-values: (****) *P* < 0.0001, ns: nonsignificant, estimated using one-way analysis of variance with Tukey's multiple comparisons test. Error bars: standard error of the mean.

To monitor trafficking of APC-dependent mRNAs, we expressed in NIH/3T3 fibroblasts a reporter carrying the β-globin coding sequence followed by 24 binding sites for the bacteriophage MS2 coat protein (MCP; Fig. 3A schematic). Binding of coexpressed MCP-GFP to these sites allows visualization and tracking of single molecules of the reporter mRNA in living cells (Fusco et al. 2003). To recapitulate the localization of APC-dependent RNAs, the reporter additionally included a control 3′UTR or the 3′UTR of *Net1* or *Rab13* (hereafter referred to as β24bs/Ctrl, β24bs/Net1, and β24bs/Rab13, respectively). As shown previously, these 3′UTR sequences are sufficient to direct peripheral distribution of this reporter transcript in NIH/3T3 cells (Moissoglu et al. 2019). We initially examined trafficking of the reporter during early stages of cell spreading, which mimic conditions in actively protruding cell regions. Indeed, live fluorescence imaging of the reporter containing the Net1 3′UTR revealed a distinct peripheral pattern after plating cells on fibronectin for 30 min (Fig. 3A; Supplemental Movie S1). Because kinesin-dependent mRNA trafficking is expected to occur on the microtubule cytoskeleton, it was important to identify microtubule-dependent events and discriminate them from other modes of motion. For this, reporter particles were tracked in cells before and after 15 min of nocodazole treatment. To identify long and linear movements, as those expected to occur on microtubules, we used two different metrics to quantitatively describe individual tracks: “Linearity of forward progression” and “Track displacement.” To determine the range of these parameters that define directed microtubule-based tracks, we compared movements before and after microtubule depolymerization. From this, we separated “long/directed” tracks (Fig. 3B). These tracks exhibit higher displacement and linearity (net displacement >4 microns, linearity >0.7), and they are absent in cells treated with nocodazole. They correspond to ∼3%–6% of the total tracks (Fig. 3B; Supplemental Movies S1, S2). Consistent with their representing persistent directed motions, this subset of tracks exhibit high Mean Square Displacements (MSD) over their lifetime (generally more than 15 µm^2^) and display positive velocity autocorrelation (Fig. 3C, left panels). In contrast, the remaining tracks, which we classify as “short/diffuse,” exhibit characteristics similar to tracks of nocodazole-treated cells. Specifically, they exhibit low MSDs and zero velocity autocorrelation, as is characteristic of diffusive Brownian motions (Fig. 3C, middle and right panels). Both “long/directed” and “short/diffuse” tracks have a similar range of lifetimes, while “long/directed” tracks have a higher mean speed (Supplemental Fig. S3).

Tracking of a reporter carrying the Rab13 3′UTR also exhibited long/directed tracks with dependence on microtubules (Fig. 3D,E). In contrast, a control reporter lacking a localizing 3′UTR did not produce tracks with these characteristics (Fig. 3D,E). Importantly, this subset of tracks was not affected by disruption of the actin cytoskeleton with cytochalasin D or following treatment with a control vehicle, DMSO (Fig. 3F; Supplemental Fig. S4; Supplemental Movies S3, S4). Thus, the reporters carrying the 3′UTR of *Net1* or *Rab13* mimic the localization pattern of APC-dependent mRNAs and allow the identification of long and linear microtubule- and 3′UTR-dependent transport events.

**FIGURE 4.**
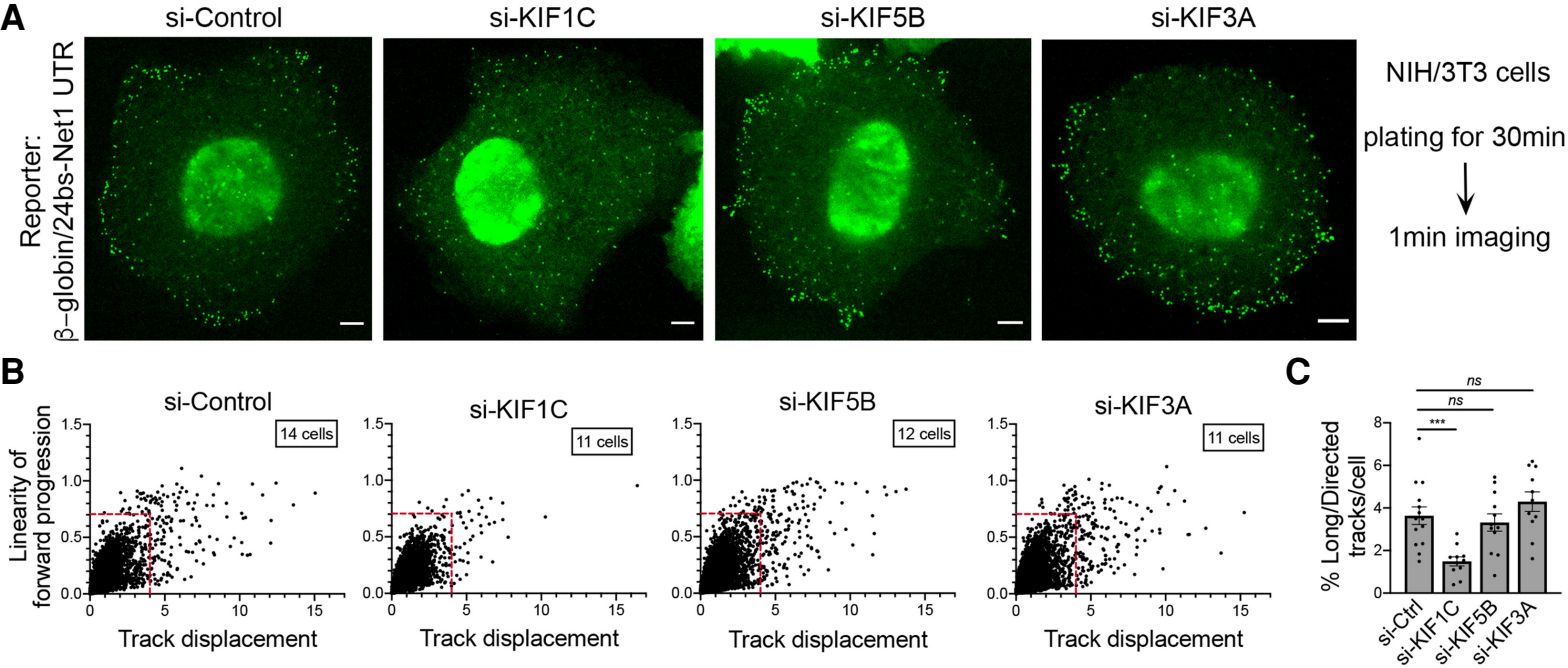
Reporter mRNAs containing the Net1 3′UTR require the Kif1c motor for long, linear microtubule-dependent displacements. (*A*) Images are snapshots of live NIH/3T3 cells taken 30 min after plating. The cells stably expressed the β24bs/Net1 mRNA reporter and MCP-GFP (Green) and were treated with the indicated siRNAs. The green spots correspond to single mRNAs detected with the MCP-GFP. High speed imaging was performed over 1 min to track individual RNA movements. See Supplemental Movies S5–S8 for time lapse imaging. Scale bars are 5 microns. (*B*) Graphs plot the displacements of individual RNA tracks (*x*-axis) over the linearity of their forward progression (*y*-axis) (defined as the mean straight line speed divided by the mean speed), using the movies of cells as shown in *A*. Red lines indicate the thresholds used to filter tracks of molecules undergoing directed movement (based on Fig. 3). (*C*) Graph depicts the percentage of directed tracks per cell following treatment with the indicated siRNAs (see panel *B*). *N* = 11–14 cells. Stars represent *P*-values: (***) *P* < 0.001, ns: nonsignificant, estimated using one-way analysis of variance with Dunnett's multiple comparisons test. Error bars: standard error of the mean.

To directly test the role of KIF1C in these trafficking events, we visualized fluorescent particles of the reporter carrying the *Net1* 3′UTR and measured the frequency of long, directed microtubule-dependent displacements in actively spreading cells following KIF1C depletion. As previously observed with endogenous transcripts (Fig. 2), reporter mRNAs became less localized when KIF1C expression was reduced with siRNAs (Fig. 4A; Supplemental Movies S5–S8). Importantly, track analysis showed that KIF1C loss significantly reduced the number of the microtubule-dependent displacements (Fig. 4B,C). To assess specificity, we depleted two additional kinesins that have been linked to APC and RNA transport, KIF5B and KIF3A (Kanai et al. 2004; Dunn et al. 2008; Cai et al. 2009; Yasuda et al. 2017; Baumann et al. 2020). Figure 4A–C shows that depleting these kinesins did not change the overall peripheral accumulation of the reporter and did not result in a reduction of the long, directed transport events. Thus, KIF1C exhibits a specific function in transporting APC-dependent mRNAs via microtubules in actively protruding cell regions.

### Peripheral clustering of APC-dependent mRNAs depends on KIF1C

**FIGURE 5.**
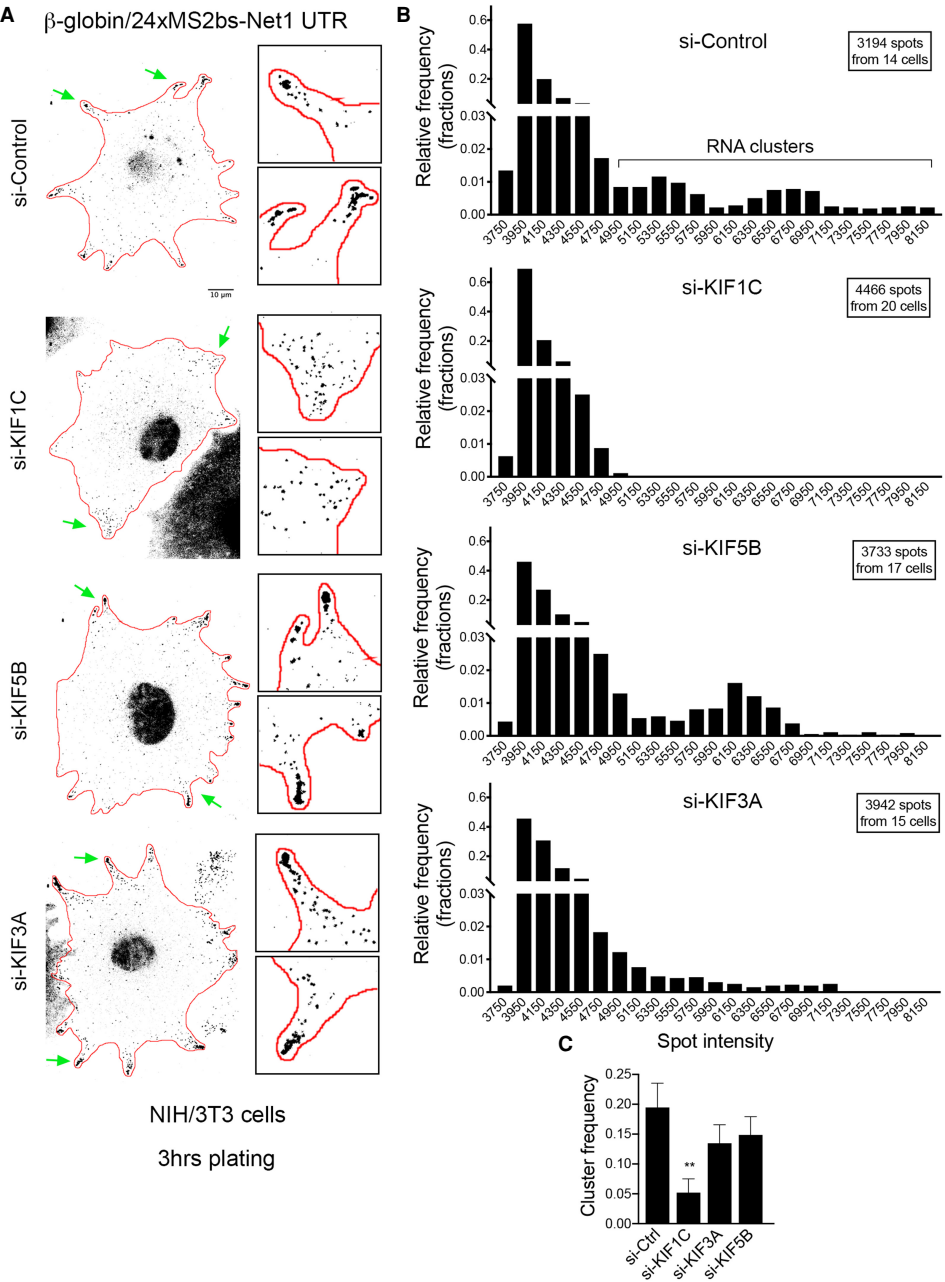
Kif1c is required for the peripheral clustering of reporter mRNAs containing the Net1 3′UTR in live mouse fibroblasts. (*A*) Images are micrographs of live NIH/3T3 cells taken 3 h after plating and expressing MCP-GFP and the β24bs/Net1 reporter mRNA. Cells were treated with the siRNAs indicated on the *left*. Scale bar is 10 microns. Boxed *insets* are magnifications of the areas indicated by green arrows. (*B*) Frequency histograms of the intensities of the β24bs/Net1 reporter mRNA spots following treatment with the indicated siRNAs, measured from images as shown in *A*, from *N* = 14–20 cells. The majority of molecules fall under a single lower intensity peak, likely indicative of single molecules, while a smaller fraction exhibits higher intensities indicative of higher order clusters. (*C*) Graph depicts the mRNA cluster frequency per cell following treatment of cells with the indicated siRNAs. Clusters correspond to β24bs/Net1 mRNA spots of intensities higher than 4950, measured from the graphs shown in *B*. *N* = 14–20 cells. Stars represent *P*-values: (**) *P* < 0.01, estimated using one-way analysis of variance with Dunnett's multiple comparisons test. Error bars: standard error of the mean.

Peripheral APC-dependent mRNAs can form large heterogeneous clusters that are translationally silent (Moissoglu et al. 2019). These clusters often associate with retracting protrusions in migrating cells, suggesting that they are part of a spatio-temporal control of protein synthesis (Moissoglu et al. 2019). Formation of these clusters is recapitulated by the reporter constructs carrying the *Net1* or *Rab13* 3′UTR, but not by a control reporter (Supplemental Fig. S5). These clusters are visible at later time points after plating (approximately 3 h), when most protrusions are not actively extending, consistent with the appearance of endogenous RNA clusters in nonextending or retracting protrusions (Fig. 5A; Moissoglu et al., 2019). These clusters can be identified as bright particles with intensities higher than those characteristic of single molecules (Fig. 5A). To test whether KIF1C is implicated in the formation of these clusters, we scored the frequency of bright particles in KIF1C-depleted cells during late stages of spreading (3 h; Fig. 5B,C; particle brightness >4950). As shown in Figure 5, while clusters formed by the *Net1* 3′UTR-reporter were readily observed in protrusions of control siRNA-treated cells, their frequency was substantially reduced, and mostly single molecules were present, when KIF1C was depleted (Fig. 5A–C). Moreover, cluster formation was only marginally affected by the depletion of KIF5B or KIF3A. Thus, KIF1C specifically controls the clustering of APC-dependent mRNAs. We note that clusters are not detected even in protrusions containing a substantial amount of single RNA molecules (see enlarged KIF1C insets in Fig. 5A), suggesting that cluster loss is not a secondary consequence of a reduced number of mRNA molecules arriving at protrusions upon KIF1C depletion. We rather think that these results indicate an additional role of KIF1C in forming higher order RNP complexes at protrusions.

### Single-molecule two-color imaging provides direct evidence that the KIF1C motor transports protrusion mRNAs

To provide direct evidence that the protrusion mRNAs are transported by the KIF1C motor, we performed two-color single-molecule imaging of mRNAs and motors, in order to visualize cotransport of the two types of molecules. To this end, we used the NIH/3T3 cells expressing the *Net1* 3′UTR-containing reporter and modified them to also stably express a KIF1C protein fused to the SunTag (KIF1C-ST_x24_), together with a single-chain variable fragment antibody fused to mScarletI (scFv-mScarletI).

**FIGURE 6.**
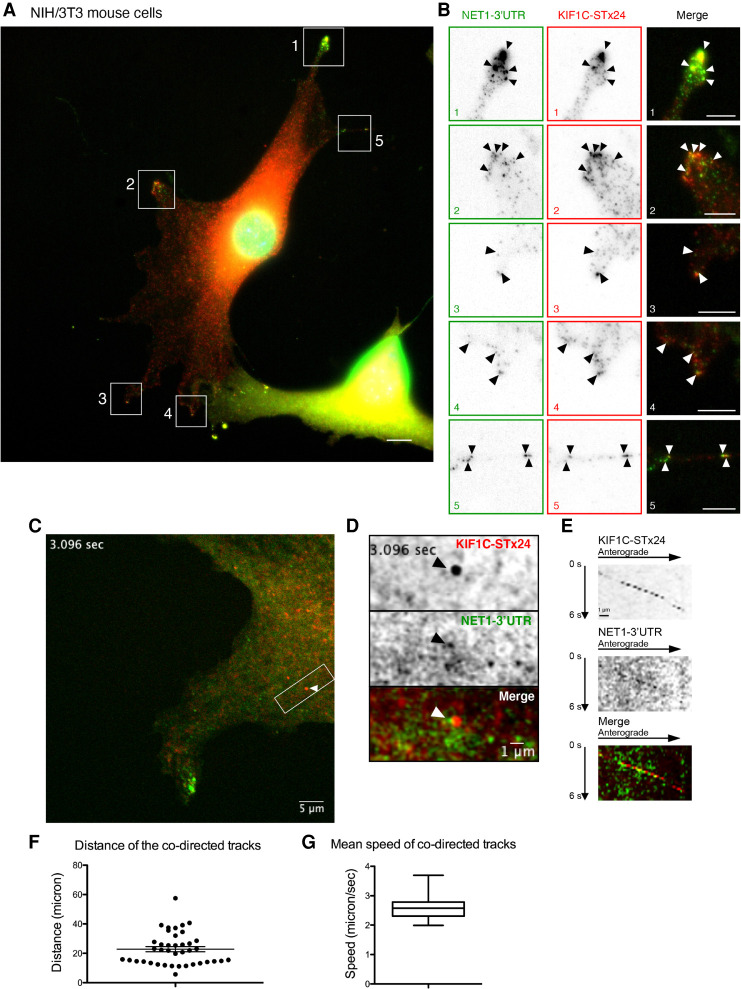
The KIF1C motor transports mRNAs containing the Net1 3′UTR to cell protrusions. (*A*) Images are micrographs of fixed NIH/3T3 cells expressing the β24bs/Net1 reporter mRNA, MCP-GFP (green), KIF1C-ST_x24_ protein, and scFv-mScarletI (red). Single molecules of the β 24bs/Net1 reporter mRNA are visible in green, while single molecules of KIF1C-ST_x24_ protein are red. The numbered white boxes are magnified in *B*. Blue: DNA stained with DAPI. Scale bar is 5 microns. (*B*) *Insets* represent magnifications of the boxed areas from the image shown in *A*. (*Left*) MCP-GFP signals labeling β24bs/Net1 mRNAs; (*middle*) scFv-mScarletI labeling KIF1C-ST_x24_ protein; (*right*) merge with mRNAs in green and KIF1C-ST_x24_ in red. Black and white arrowheads indicate colocalization of single molecules of β24bs/Net1 mRNAs and KIF1C-ST_x24_. Scale bar is 5 microns. (*C*) Snapshot of a live NIH/3T3 cell expressing β24bs/Net1 mRNA, MCP-GFP (green), KIF1C-ST_x24_ protein, and scFv-mScarletI (red). Snapshot is extracted from Supplemental Movie S9. The white arrowhead indicates a cotransport event of a single molecule of β24bs/Net1 mRNA (green) with a KIF1C-ST_x24_ protein (red). The boxed area is magnified in panels *D* and *E*. Scale bar is 5 microns. (*D*) Magnification of the boxed area in panel *C*, highlighting a cotransport event. (*Top*) KIF1C-ST_x24_; (*middle*) β24bs/Net1 mRNA; (*bottom*) merged panel with the β24bs/Net1 mRNA in green and KIF1C-ST_x24_ in red. Scale bar is 1 micron. (*E*) Kymograph from Supplemental Movie S9, showing the trajectory of a single molecule of KIF1C-ST_x24_ (*top* panel), a single molecule of β24bs/Net1 mRNA (*middle* panel), and the merge (*bottom* panel). The cellular area shown corresponds to panel *D*. (*F*) The graph depicts the distance traveled by cotransported molecules of KIF1C-ST_x24_ and β24bs/Net1 mRNA. Each data point is a track (40 tracks in total), and the mean and 95% confidence intervals are shown by *horizontal* lines. Source data are provided in Supplemental Table S4. (*G*) Boxplot depicting the mean speed of cotransported molecules of KIF1C-ST_x24_ and β24bs/Net1 mRNA in NIH/3T3 cells. Speed is microns/second. The *vertical* bars display the first and last quartile, the box corresponds to the second and third quartiles, and the *horizontal* line to the mean (40 tracks in total). Source data are provided in Supplemental Table S4.

Imaging of fixed cells showed that the KIF1C-SunTag_x24_ motor and reporter mRNAs accumulated in protrusions as expected (Fig. 6A,B; Supplemental Fig. S4A,B). Moreover, we could also occasionally detect colocalization events where a single molecule of KIF1C-SunTag_x24_ would colocalize with a single reporter mRNA at internal cellular areas. To confirm that this colocalization was relevant to mRNA transport, we performed two-color live-cell imaging using movies recorded at a high frame rate (7.36 fps for 52 sec). This allowed the detection of cotransport events, in which a single molecule of KIF1C-ST_x24_ moved with a reporter mRNA molecule in a rectilinear manner at high speed (Fig. 6C,D; Supplemental Movie S9; Supplemental Fig. S4C,D; Supplemental Movie S10). Kymographs confirmed that both molecules moved together in an anterograde direction toward protrusion (Fig. 6E; Supplemental Fig. S4E), traveling an average distance of 22 microns at speeds of 2.6 µm/sec (Fig. 6F,G). Taken together, these data demonstrate that the KIF1C kinesin actively transports this *Net1* reporter mRNA to cell protrusions along microtubule cables.

## DISCUSSION

RNA transport along the cytoskeleton is a well-established mechanism allowing subcellular mRNA localization and local translation. In mammals, a large class of mRNAs localize to cytoplasmic protrusions of many cell types, where they are anchored at the plus-end of detyrosinated microtubules by APC. Here, we show that these mRNAs associate with the microtubule motor KIF1C and interestingly, they include the KIF1C mRNA itself. We show that APC-dependent mRNAs and *KIF1C* protein colocalize in protrusions and can also be seen cotransported together along directed tracks. Moreover, the peripheral localization of these mRNAs as well as their microtubule-dependent motion depend on KIF1C, demonstrating that it is an essential motor that transports APC-dependent mRNAs to protrusions. Our data provide a striking in vivo visualization of the cotransport of individual RNA molecules with a specific molecular motor, involved in a widespread RNA transport pathway.

### The kinesin KIF1C controls a widespread mammalian mRNA transport system

RNA localization controls spatial and temporal aspects of gene expression in a variety of species and cell types. Although its significance is better understood in specialized cells such as neurons, recent reports highlight its widespread prevalence, including in cells with a mesenchymal phenotype (Mardakheh et al. 2015; Wang et al. 2017; Fazal et al. 2019; Chouaib et al. 2020; Costa et al. 2020). Our current understanding of the transport mechanisms includes the requirement of *cis*-acting sequence elements and *trans*-acting factors, which work with the actin or microtubule networks and motor proteins to bring mRNAs to their destination (Medioni et al. 2012; Cody et al. 2013; Bovaird et al. 2018). Nevertheless, our knowledge of common RNA transport mechanisms that operate in most, if not all cells, is limited. In the case of protrusion mRNAs, which localize in all cell types examined so far, their localization was shown to require APC and detyrosinated microtubules (Mili et al. 2008; Wang et al. 2017). It is not yet known whether APC is transported together with the KIF1C–RNA complexes or whether it is independently transported and subsequently associated with RNAs at the periphery. In vitro studies suggest that motile complexes can be formed between mRNA, APC and KIF3A (Baumann et al. 2020). Our data, however, clearly show the involvement of KIF1C in transporting protrusion mRNAs in vivo. Moreover, APC was shown to accumulate at the leading edge of migrating cells using kinesin-1 and kinesin-2 (Mimori-Kiyosue et al. 2000; Nakamura et al. 2001; Ruane et al. 2016). The use of distinct motors suggests an independent transport for APC and protrusion mRNAs. It is also possible that this diversity of motors reflects differences between cell types, as has been described for the β-actin RNA that uses different motors in neurons and fibroblasts (see Introduction; Latham et al. 2001; Ma et al. 2011; Nalavadi et al. 2012; Song et al. 2015). One potential reason for using different motors could pertain to the dynamics and transport speeds required in each case. In this context, the fact that KIF1C appears to be the fastest human cargo transporter (Lipka et al. 2016) might provide an advantage that could underlie its preferential use in the highly dynamic mesenchymal cells. Furthermore, kinesins other than KIF1C, such as KIF5B, contribute to localization of APC-dependent RNAs (Yasuda et al. 2017). We speculate that they may function in specialized cells or affect different aspects of localization that are distinct from transport per se. In line with this idea, protrusion mRNAs display a complex translational regulation concomitant to the protrusion dynamics and a local reorganization of RNA clusters (Moissoglu et al. 2019), likely requiring a number of yet uncharacterized actors.

Another important question deals with the adaptors that link KIF1C to mRNAs. On one hand, proteomic data showed that KIF1C protein interacts with the exon junction complex (EJC; Hein et al. 2015). The EJC is assembled on spliced RNAs and serves as an interaction platform for proteins that direct mRNA export, localization, translation and nonsense-mediated mRNA decay (NMD), suggesting a role for the EJC in the transport of protrusion mRNAs. On the other hand, regions rich in G and A nucleotides are present in the 3′UTR of APC-dependent RNAs and are part of the localization element (Costa et al. 2020; Moissoglu et al. 2020). Furthermore, the 3′UTR is sufficient to direct protrusion localization of intronless exogenous constructs (Moissoglu et al. 2019, 2020), indicating that a potential KIF1C recruitment through exon–exon junctions might not be necessary for protrusion localization. Specific RNA-binding proteins could serve as adaptors that mediate KIF1C recruitment similar to the model suggested for other RNA transport complexes. However, given that high-throughput mRNA–protein cross-linking approaches previously showed that KIF1C directly binds mRNAs (Baltz et al. 2012; Castello et al. 2012), an alternative interesting possibility would be that in this case the motor directly selects and binds to its RNA cargo.

### KIF1C triggers mRNA clustering in cytoplasmic protrusions

Our live imaging experiments show that reporter mRNAs are transported predominantly as single molecules during the early stages of cell spreading, reminiscing the single-molecule appearance of *RAB13* mRNA in actively protruding regions in migrating cells (Moissoglu et al. 2019). Remarkably, APC-dependent mRNAs coalesce into higher order clusters at peripheral regions during later time points of cell spreading, and this phenotype depends specifically on KIF1C. We think that clustering is not merely a consequence of peripheral mRNA accumulation, because we have not observed it in the absence of KIF1C even at protrusions still containing substantial numbers of single mRNA molecules. We rather favor the explanation that clustering is a separate function of KIF1C that is temporally and spatially regulated. Given that these clusters contain stably anchored mRNAs (Mili et al. 2008), we envision that KIF1C switches from a microtubule motor to an mRNA anchoring module promoting clustering. A similar switch has been observed in Drosophila oocytes, whereby Dynein converts from a motor of *gurken* mRNA to a static anchor at its final destination (Delanoue et al. 2007). Such a switch on KIF1C may take place on pre-existing motor molecules as they reach the periphery or may be a function of newly synthesized KIF1C translated from its peripherally localized mRNA. It is still unclear how such a switch would occur and/or whether it might additionally involve a change in the RNA-binding mode of KIF1C (direct or indirect through other RNA-binding proteins). Clusters of APC-dependent mRNAs have been previously reported to be heterogeneous and to contain translationally silent mRNAs (Moissoglu et al. 2019). Thus, overall our results point to a spatially and temporally controlled mRNA clustering role of KIF1C that is separate from its motor function and that might be coordinated with translational regulation.

### RNA transported by KIF1C mediates diverse functions at cell protrusions

Peripheral localization of APC-dependent RNAs promotes cell migration (Wang et al. 2017). Specifically, approaches targeting the localization elements of these mRNAs, as a group (Wang et al. 2017) or individually (Moissoglu et al. 2020), resulted in inhibition of cell migration. These effects are likely due to a requirement for locally translating these mRNAs for full activation of the encoded proteins (Moissoglu et al. 2020). Interestingly, KIF1C has been shown to control adhesion dynamics and cell migration (Theisen et al. 2012). It was proposed to act via the trafficking of α5β1 integrins. While we cannot completely rule out that KIF1C indirectly affects peripheral RNA localization through altering adhesion dynamics, we consider this highly unlikely given the physical interaction and cotransport of KIF1C and RNAs that we report here. Instead, our results indicate that the transport of APC-dependent mRNAs to the periphery by KIF1C likely itself contributes to the mechanism by which this kinesin controls cell migration. Along this line, the GO terms associated with the top 200 KIF1C-associated mRNAs presented in this study (i.e., organelle organization; plasma membrane bounded cell projection organization; microtubule-based transport; cilium organization; Supplemental Table S5) indicate how KIF1C-mediated mRNA transport could impact processes related to cell motility.

### KIF1C protein localizes its own mRNAs to cell protrusions: a transport feedback loop?

The *KIF1C* transcript localizes to cytoplasmic protrusions in mammalian cells. Moreover, it colocalizes with KIF1C protein in protrusions (Chouaib et al. 2020), and we show here that the KIF1C protein physically associates with its own mRNA. This local accumulation of KIF1C could be involved in an RNA clustering and anchoring mechanism as discussed above, but it could also serve to transport additional mRNAs by alternating back-and-forth movements on the cytoskeleton. Indeed, locally translated KIF1C protein would allow the motor to explore the local cytoplasm and transport back additional mRNAs to protrusions using the same MT tracks. Such a bidirectional motility has been reported for KIF1C and it is mediated by the scaffold protein Hook3. This protein forms a complex between dynein and KIF1C (Kendrick et al. 2019), and regulates their activities to allow the motor to perform multiple transport cycles while avoiding a tug-of-war between opposite motors (Siddiqui et al. 2019). The fact that KIF1C also brings its own mRNA to protrusions suggests the possible existence of a positive feedback loop in which locally translated KIF1C provides additional motor molecules to sustain the persistent and directional transport of its RNA cargoes, to locally maintain protrusive extensions during cell movement.

## MATERIALS AND METHODS

### Generation and maintenance of cell lines

The HeLa-Kyoto cells stably transfected with the KIF1C-GFP BAC were previously described (Poser et al. 2008; Maliga et al. 2013; Chouaib et al. 2020). HeLa Flp-in H9 (a kind gift of S. Emiliani) and the BAC-GFP cells were maintained in Dulbecco's Modified Eagle Medium (DMEM, Gibco) supplemented with 10% fetal bovine serum (FBS, Sigma), 100 U/mL penicillin/streptomycin (Sigma) and with 400 µg/mL G418 (Gibco) for the HeLa-Kyoto KIF1C-GFP tagged BAC cells. NIH/3T3 cells were maintained in DMEM supplemented with 10% calf serum, sodium pyruvate and penicillin/streptomycin at 37°C, 5% CO_2_. MDA-MB-231 human breast cancer cells (ATCC) were grown in Leibovitz's L15 media supplemented with 10% fetal bovine serum and penicillin/streptomycin at 37°C in atmospheric air. Stable HeLa cell lines expressing a KIF1C-GFP cDNA were created using the Flp-in system in HeLa H9 cells. Flp-in integrants were selected on hygromycin (150 µg mL^−1^). To generate cell lines expressing RNA reporters, NIH/3T3 cells were infected with lentivirus expressing tdMCP-GFP (Addgene plasmid #40649), and GFP-expressing cells with a low level of GFP expression were sorted by FACS. This stable population was infected with pInducer20-based reporter constructs expressing β-globin followed by 24xMS2 binding sites and the mouse Net1, Rab13, or control 3′UTRs (pIND20-β24bs/Net1 3′UTR; pIND20-β24bs/Rab13 3′UTR; pIND20-β24bs/Ctrl 3′UTR; Moissoglu et al, 2019). Stable lines were selected with geneticin (Thermo Fisher Scientific) and expression of the reporter was induced by addition of 1 µg/mL doxycycline (Fisher Scientific) 2–3 h before imaging.

For the two-color tracking experiment, NIH/3T3 cells expressing the pIND20-β24bs/Net1 reporter and tdMCP-GFP described below were modified as follows. Stable expression of scFv-mScarletI-GB1 in NIH/3T3 cells was achieved by lentiviral-mediated integration of a plasmid derived from Addgene (#60906) and sorted by FACS with a low level of mScarletI expression. Then a lentivirus expressing a KIF1C fusion with 24 repeats of the GCN4 peptide array was used to infect these NIH/3T3 cells allowing the detection of KIF1C-STx24 protein with scFv-mScarletI-GB1.

### Treatments with siRNAs and drugs

HeLa cells were seeded on 0.17 mm glass coverslips deposited in six-well plates. Cells were transfected at 70% confluency using JetPrime (Polyplus). Double-stranded siRNAs (30 pmoles) were diluted into 200 µL of JetPrime buffer. JetPrime reagent was added (4 µL) and the mixture was vortexed. After 10 min at room temperature (RT), it was added to the cells grown in 2 mL of serum-containing medium. After 24 h, the transfection medium was replaced with fresh growth medium and cells were fixed 24 h later. The sequences of the siRNA were: KIF1C: 5′-CCCAUGCCGUCUUUACCAUdCdG; control: 5′-CAACAGAAGGAGAGCGAAAdTdT. For knockdown of human or mouse Kif1c the following additional siRNAs were used: Hs_KIF1C_5 (Qiagen cat# SI02655401), Hs_KIF1C_6 (Qiagen cat# SI02781331), Hs_KIF1C_7 (Qiagen cat# SI02781338), Hs_KIF1C_8 (Qiagen cat# SI03019744), Mm_Kif1c_2 (Qiagen cat# SI00239687), Mm_Kif1c_3 siRNA (Qiagen cat# SI00239694), and AllStars negative control siRNA (Qiagen cat# 1027281). siRNAs were delivered into cells using Lipofectamine RNAiMAX (Thermo Fisher Scientific, cat# 13778-150) according to the manufacturer's instructions. Cells were assayed 72 h after siRNA transfection.

For drug treatments, 10 µM nocodazole or Cytochalasin D, or an equal volume of DMSO, were added to the growth media for 15 min.

### Plasmid construction

Plasmids were generated with standard molecular biology techniques. The GFP-APC plasmid was a gift from Inke Nathke. The KIF1C-mCherry plasmid was a gift from Anne Straube (Addgene plasmid #130978). The inducible constructs expressing MS2-containing RNA reporters (pIND20-β24bs/Net1 3′UTR; pIND20- β24bs/Rab13 3′UTR) are described in Moissoglu et al. (2019). They contain the human β-globin gene followed by 24xMS2 binding sites and the mouse Net1, Rab13, or control 3′UTRs. The control reporter (pIND20-β24bs/Ctrl 3′UTR) carries a short, random, vector-derived UTR sequence. To generate the KIF1C-SunTagx24 cell lines, the KIF1C cDNA was amplified by PCR and cloned in pHRdSV40-K560-GCN4 × 24 (Addgene #72229), in place of the K560 cDNA and upstream of the SunTag. Then the KIF1C-STx24 sequence was PCR amplified and cloned into pHAGE-Ubc-MCP-YFP (Addgene #31230) in place of the MCP-GFP sequence. Maps and sequences are available upon request.

### Immunoprecipitation and microarrays

HeLa cells containing the KIF1C-GFP BAC were grown to near confluence in 10 cm plates, and two plates were used per IP. Cells were rinsed in ice-cold PBS, and all subsequent manipulations were performed at 4°C. Cells were scraped in HTNG buffer (20 mM HEPES-KOH pH 7.9, 150 mM NaCl, 1% Triton X-100, 10% glycerol, 1 mM MgCl_2_, 1 mM EGTA), containing an antiprotease cocktail (Roche Diagnostic). Cells were incubated for 20 min on a rotating wheel, and cellular debris were removed by centrifugating the extracts 10 min at 20,000*g*. Beads coated with GFP-trap antibody (ChromoTek), or uncoated as control, were washed in HNTG (25 µL of beads per IP). Beads were incubated 1 h with a control extract to saturate nonspecific binding and then incubated with the proper extract. After 4 h of incubation on a rotating wheel, beads were washed four times in HNGT with anti-protease, and twice with PBS. Beads were then incubated with TRIzol to extract RNAs, and RNA purification was done as recommended by the manufacturer. The resulting RNAs were amplified and converted into cDNAs by the WT PICO kit (Thermo Fisher), and hybridized on an HTA 2.0 chip on an Affymetrix platform (Thermo Fisher). Experiments were performed in duplicates, data were normalized and averaged. Data are deposited on GEO with the following accession number: GSE161316.

For GFP-APC immunoprecipitation, cells were lysed with a buffer containing 50 mM Tris pH 7.4, 1% Triton X-100, 75 mM NaCl, 10 mM MgCl_2_, 10% glycerol and a cocktail of protease and phosphatase inhibitors (Thermo Fischer Scientific, cat# 1861281). Lysates were cleared by centrifugation and mixed with GFP-Trap Magnetic Agarose beads (Chromotek, cat# gtma-10) for 1.5 h, at 4°C. Immobilized complexes were eluted with Laemmli's buffer and analyzed by SDS-PAGE and immunoblotting.

### Western blot

For western blot detection the following antibodies were used: anti-GFP rabbit polyclonal (Invitrogen, cat# A11122, 1/2000 dilution), anti-KIF1C rabbit polyclonal (Proteintech, cat# 12760-1-AP), anti-KIF1C rabbit polyclonal (Bethyl Laboratories, cat# A301-070A, 1/2000 dilution), anti-α-tubulin mouse monoclonal (Sigma-Aldrich, cat# T6199, 1/10,000 dilution), anti-mCherry mouse monoclonal [1C51] (Abcam, cat# ab125096, 1/2000 dilution).

### RNA analyses

For total RNA analysis, cells were lysed with TRIzol LS reagent (Thermo Fisher Scientific, cat# 10296010) and RNA was extracted according to the manufacturer's instructions. Isolated RNA was treated with RQ1 RNase-free DNase (M6101, Promega) and analyzed with the nCounter system (NanoString Technologies) using a custom-made codeset. Data were processed using nSolver analysis software (NanoString technologies).

### Single-molecule FISH

Cells grown on glass coverslips were fixed for 20 min at RT with 4% paraformaldehyde diluted in PBS, and permeabilized with 70% ethanol overnight at 4°C. For smFISH, we used a set of 44 amino-modified oligonucleotide probes against the GFP-IRES-Neo sequence (sequences given in Supplemental Table S2). Each oligonucleotide probe contained 4 primary amines that were conjugated to Cy3 using the Mono-Reactive Dye Pack (PA23001, GE Healthcare Life Sciences). To this end, the oligos were precipitated with ethanol and resuspended in water. For labeling, 4 µg of each probe was incubated with 6 µL of Cy3 (1/5 of a vial resuspended in 30 µL of DMSO), and 14 µL of carbonate buffer 0.1 M pH 8.8, overnight at RT and in the dark, after extensive vortexing. The next day, 10 µg of yeast tRNAs were added and the probes were precipitated several times with ethanol until the supernatant lost its pink color. For hybridization, fixed cells were washed with PBS and hybridization buffer (15% formamide in 1xSSC), and then incubated overnight at 37°C in the hybridization buffer also containing 130 ng of the probe set for 100 µL of final volume, 0.34 mg/mL tRNA (Sigma), 2 mM VRC (Sigma), 0.2 mg/mL RNase-free BSA (Roche Diagnostic), and 10% Dextran sulfate. The next day, the samples were washed twice for 30 min in the hybridization buffer at 37°C, and rinsed in PBS. Coverslips were then mounted using Vectashield containing DAPI (Vector laboratories, Inc.).

For smiFISH (Tsanov et al. 2016), 24 to 48 unlabeled primary probes were used (sequences given in Supplemental Table S2). In addition to hybridizing to their targets, these probes contained a FLAP sequence that was hybridized to a secondary fluorescent oligonucleotide. To this end, 40 pmoles of primary probes were prehybridized to 50 pmoles of secondary probe in 10 µL of 100 mM NaCl, 50 mM Tris-HCl, 10 mM MgCl_2_, pH 7.9. Hybridization was performed at 85°C for 3 min, 65°C for 3 min, and 25°C for 5 min. The final hybridization mixture contained the probe duplexes (2 µL per 100 µL of final volume), with 1× SSC, 0.34 mg/mL tRNA (Sigma), 15% Formamide, 2 mM VRC (Sigma), 0.2 mg/mL RNase-free BSA, 10% Dextran sulfate. Slides were then processed as above. For Supplemental Figure S1A, the probes used were RNA and not DNA (sequence in Supplemental Table S2). The protocol was similar except that hybridization was performed at 48°C and that 50 ng of the primary probe (total amount of the pool of probes) and 30 ng of the secondary probes were used per 100 µL of hybridization mix.

For FISH of mouse cells, cells plated on fibronectin-coated coverslips were fixed for 20 min at RT with 4% paraformaldehyde in PBS. FISH was performed with the ViewRNA ISH Cell Assay kit (Thermo Fisher Scientific) according to the manufacturer's instructions. The flowing probe sets were used: Kif1c (cat# VB6-3200442), Net1 (cat# VB1-3034209), Rab13 (cat# VB1-14374), Ddr2 (cat# VB1-14375), Dynll2 (cat# VB1-18646), Cyb5r3 (cat# VB1-18647). To detect polyA+ RNAs, LNA modified oligodT probes (30 nt) labeled with ATTO 655 were added during hybridization, preamplification, amplification, and last hybridization steps of the ViewRNA ISH Cell Assay. Cell mask stain (Thermo Fisher Scientific) was used to identify the cell outlines. Samples were mounted with ProLong Gold antifade reagent (Thermo Scientific)

### Imaging of fixed cells

Microscopy slides were imaged on a Zeiss Axioimager Z1 wide-field microscope equipped with a motorized stage, a camera scMOS ZYLA 4.2 MP, using a 63× or 100× objective (Plan Apochromat; 1.4 NA; oil). Images were taken as *z*-stacks with one plane every 0.3 µm. The microscope was controlled by MetaMorph and figures were constructed using ImageJ, Adobe Photoshop and Illustrator. For the small smiFISH screen, 96-well plates were imaged on an Opera Phenix High-Content Screening System (PerkinElmer), with a 63× water-immersion objective (NA 1.15). Three-dimensional images were acquired, consisting of 35 slices with a spacing of 0.3 µm. FISH images of mouse cells were obtained using a Leica SP8 confocal microscope, equipped with a HC PL APO 63× CS2 objective. *Z*-stacks through the cell volume were obtained and maximum intensity projections were used for subsequent analysis.

### Image analysis and quantifications

Automated nuclear and cell segmentation was performed with a custom algorithm based on the U-net deep convolutional network (Ronneberger et al. 2015). Nuclear segmentation was performed with the DAPI channel; cell segmentation was performed with the autofluorescence of the actual smFISH image. For segmentation, 3D images were projected into 2D images as described previously (Tsanov et al. 2016). Messenger RNAs were detected with FISH-quant (Mueller et al. 2013) by applying a local maximum detection on LoG filtered images. For calculation of Peripheral Distribution Index (PDI) a custom Matlab script was used. The code is described and is available in Stueland et al. (2019).

### Imaging of live cells

Live imaging (for dual visualization of β24bs/Net1 reporter RNA and KIF1C-ST_x24_ protein) was done using a spinning disk confocal microscope (Nikon Ti with a Yokogawa CSU-X1 head) operated by the Andor iQ3 software. Acquisitions were performed using a 100× objective (CF1 PlanApo λ 1.45 NA oil), and an EMCCD iXon897 camera (Andor). For two-color imaging, samples were sequentially excited at 488 and 540 nm. We imaged at a rate of 7.36 fps for 52 sec. The power of illuminating light and the exposure time were set to the lowest values that still allowed visualization of the signal. This minimized bleaching, toxicity and maximized the number of frames that were collected. Cells were maintained in anti-bleaching live cell visualization medium (DMEM^gfp^; Evrogen), supplemented with 10% fetal bovine serum at 37°C in 5% CO_2_ and rutin at a final concentration of 20 mg/L.

Live imaging (for β24bs/Net1 reporter RNA tracking) was done using a Nikon Eclipse Ti2-E inverted microscope, equipped with a motorized stage, a Yokogawa CSU-X1 spinning disk confocal scanner unit, and operated using NIS-elements software. Acquisitions were performed using an Apochromat TIRF 100× oil immersion objective (N.A. 1.49, W.D. 0.12 mm, F.O.V. 22 mm) and a Photometrics Prime 95B Back-illuminated sCMOS camera with W-view Gemini Image splitter. Constant 37°C temperature and 5% CO_2_ were maintained using a Tokai Hit incubation system. Cells were plated on fibronectin (2 mg/mL)-coated 35 mm glass bottom dishes, and samples were excited using a 488 nm (20 mw) laser line and imaged at a rate of 6.66 fps for 60 sec.

### Live cell imaging quantification

Images were processed for brightness/contrast, cropped and annotated using ImageJ/FIJI. Kymographs were generated using standard ImageJ/Fiji plugins. Film presentation in figures and videos were edited in ImageJ/Fiji. Bicolor tracking of KIF1C-STx24 proteins and β24bs/Net1 mRNAs was performed using the Manual Tracking plugin in ImageJ/Fiji.

Single color tracking of β24bs/Net1 mRNAs was performed using TrackMate plugin in ImageJ/Fiji. For every cell, all tracks lasting for >2.5 sec (approximately 17 consecutive frames) were used for analysis. Values of “Track displacement,” “Linearity of forward progression,” “track duration,” and “mean speed” were extracted and plotted. “Track displacement” is defined as the distance from the first to the last spot of the track. “Linearity of forward progression” is the mean straight line speed divided by the mean speed; where mean straight line speed is defined as the net displacement divided by the total track time. MSD and velocity autocorrelation of tracks was determined using MSDanalyzer (https://github.com/tinevez/msdanalyzer). For RNA cluster analysis, TrackMate was used to identify spots and extract intensity values. Frequency histograms of spot intensities were plotted using GraphPad Prism software.

## SUPPLEMENTAL MATERIAL

Supplemental material is available for this article.

## Supplementary Material

Supplemental Material
