# Supplementary material for: The kinesin KIF1C transports APC-dependent mRNAs to cell protrusions
Source: RNA. 2021 Dec;27(12):1528–44. doi: 10.1261/rna.078576.120 (PMC8594469; doi:10.1261/rna.078576.120)
Supplement: Supplemental Material [file supp_27_12_1528__DC1.html]

The kinesin KIF1C transports APC-dependent mRNAs to cell protrusions — Supplemental Material 

# The kinesin KIF1C transports APC-dependent mRNAs to cell protrusions

## Supplemental Material

- Supplemental\_Figure\_Legends.docx
- Supplemental\_Figures.pdf
- Supplemental\_Movie\_S1.mp4
- Supplemental\_Movie\_S2.mp4
- Supplemental\_Movie\_S3.mp4
- Supplemental\_Movie\_S4.mp4
- Supplemental\_Movie\_S5.mp4
- Supplemental\_Movie\_S6.mp4
- Supplemental\_Movie\_S7.mp4
- Supplemental\_Movie\_S8.mp4
- Supplemental\_Movie\_S9.mp4
- Supplemental\_Movie\_S10.mp4
- Supplemental\_Table\_S1.xlsx
- Supplemental\_Table\_S2.xlsx
- Supplemental\_Table\_S3.xlsx
- Supplemental\_Table\_S4.xlsx
- Supplemental\_Table\_S5.xlsx
