# Supplementary material for: The kinesin KIF1C transports APC-dependent mRNAs to cell protrusions
Source: RNA. 2021 Dec;27(12):1528–44. doi: 10.1261/rna.078576.120 (PMC8594469; doi:10.1261/rna.078576.120)
Supplement: Supplemental Material [file supp_078576.120_Supplemental_Figure_Legends.docx]

**Figure S1: Identification of mRNAs associated with the KIF1C motor.**

**A**- Localization of mRNAs in protrusion of human cells. Images are micrographs of HeLa cells labelled by smiFISH with probes against endogenous genes. Top: Cy3 fluorescent signals corresponding to endogenous KIF1C, NET1, TRAK2 and RAB13 mRNAs. Bottom: merge panel with red being the Cy3 fluorescent smiFISH signals and blue corresponding to DNA stained with DAPI. Scale bar: 10 microns. Arrowheads indicate accumulation of single molecule mRNA at cell protrusions.

**B**- The KIF1C motor preferentially associates with APC-dependent mRNAs. Correlation analysis of mRNA enrichment in protrusions (x axis; Ps/CB is the Protrusion versus Cell Body ratio; data from Wang et al., 2017) versus the enrichment fold of the same mRNAs in KIF1C-GFP IP (y-axis; KIF1C-GFP versus control IP). Red dots depict APC-dependent mRNAs, green dots depict ribosomal protein mRNAs (APC-independent mRNAs), based on data from Wang et al., 2017. See Table S3 for source data.

**C**-**E** Single molecule colocalization of KIF1C-ST-x24 with NET1 mRNAs. Images are micrographs of Hela cells stably expressing KIF1C-STx24 and scFv-GFP. Red: Cy3 fluorescent signals corresponding to mRNAs labeled by smiFISH with probes against endogenous NET1 mRNA (D), or CRM1 (C) and RPB1 (E) as controls. Green: GFP signal corresponding to KIF1C motor protein. Blue: DNA stained with DAPI. Scale bar: 10 microns in the main panels, 2.5 microns in the zoom. Arrowheads: single mRNA molecules.

**Figure S2: KIF1C is required for the localization of APC-dependent mRNAs to cytoplasmic protrusions in mouse and human cells.**

**A**- Efficiency of the KIF1C knock-down measured by smiFISH. The graph depicts the number of single molecules of KIF1C mRNA per HeLa cell, following treatment with control siRNA (orange bar) or KIF1C siRNA (blue bar). Error bars: standard deviation (n=2); number of cells: 50 per condition.

**B**- The KIF1C motor is required for accumulation of RAB13 mRNA in protrusions of HeLa cells. Images are micrographs of HeLa cells labelled by smiFISH with probes against RAB13 mRNAs. Left: Hela cells treated with a control siRNA; right: HeLa cells treated with siRNA against KIF1C. Top: Cy3 fluorescent signal corresponding to endogenous RAB13 mRNA. Lower panels are the merge with Cy3 fluorescent signals in red and DNA in blue (DAPI staining). Scale bar: 10 microns. The orange arrow indicates a protrusion accumulating RAB13 mRNA.

**C**- Quantification of NET1 or RAB13 mRNA localization in HeLa cells treated with the indicated siRNAs against KIF1C. Bars represent the mean and error bars the 95% confidence interval. Points indicate individual cells (N=30) observed in two independent experiments.

**D**- Treatment of MDA-MB-231 cells with the indicated siRNAs against KIF1C. Left panel: Western blot indicating the efficiency of KIF1C knockdown. Right graphs: Quantification of NET1 or RAB13 mRNA localization. Bars represent the mean and error bars the 95% confidence interval. Points indicate individual cells (N=30) observed in two independent experiments.

**E**- Micrographs of NIH/3T3 cells labelled by smFISH with probes against the indicated mRNAs. Cells were treated with Kif1C or Control siRNAs. Green outline: cell contour; blue outline: DAPI staining; black spots: mRNAs detected by smFISH. Scale bars: 10 microns.

**F**- Intracellular distribution of *Cyb5r3* mRNA calculated by their PDI values, after treatment with Control or Kif1C siRNAs. Horizontal lines represent the mean with 95% confidence interval. Points indicate individual cells. *p*<0.0001, estimated by Student’s t-test.

**G**-Localization of APC-independent RNAs is not affected by Kif1c depletion in NIH/3T3 fibroblasts. The graph plots PDI values of *Ddr2*, *Rps20*, *Rpl27a* and polyA+ RNAs, measured from smFISH images of cells treated with Control or Kif1c siRNAs. Points indicate individual cells. Stars are p-values: ****<0.0001, estimated by analysis of variance with Tukey’s multiple comparisons test.

**H**- Expression of localized (APC-dependent and -independent) and non-localized mRNAs is not affected by Kif1c depletion in NIH/3T3 fibroblasts. The graph depicts Log2 RNA expression ratios (Kif1c depleted versus control cells), measured by Nanostring analysis. n=3. Bars indicate mean +/- standard error.

**Figure S3: Analysis of single molecule RNA tracks**

**A**- Frequency histogram of track duration of long/directed or short/diffuse tracks. Analyzed tracks correspond to tracks of the β24bs/Net1 reporter RNA from untreated cells presented in Figure 3A-C.

**B**- Frequency histogram of mean track speed of the same tracks analyzed in A.

**Figure S4: Long/directed tracks of the β24bs/Net1 reporter RNA do not require the actin cytoskeleton**

Images are snapshots of live NIH/3T3 cells taken 30 minutes after plating. High speed imaging was performed over 1min to track individual RNA movements before and after 15 minutes of Cytochalasin D or vehicle control (DMSO) as indicated. See movies S3 and S4 for time lapse imaging. The cells stably expressed the β24bs/Net1 reporter mRNA and MCP-GFP (Green). Scale bar: 5 microns. The associated graphs plot the displacements of individual tracks (x-axis) over the linearity of their forward progression (y-axis), (defined as the mean straight line speed divided by the mean speed). Red lines indicate the thresholds used to filter tracks of molecules undergoing directed movement. N=6-7 cells.

**Figure S5: Reporter mRNAs containing Net1 and Rab13 3’UTRs form clusters at the tips of protrusions in mouse fibroblasts**

Micrographs of live NIH/3T3 cells taken 3h after plating and expressing MCP-GFP and the β24bs reporter mRNA carrying the indicated 3’UTRs. Red line: cell contour. Scale bars are 10 microns. Note that reporter mRNAs containing the Net1 or Rab13 3’UTR accumulate in clusters at the tips of protrusions, while the control reporter does not. Boxed insets (right) are magnifications of the areas indicated by the green arrows.

**Figure S6: Reporter mRNAs containing the Net1 3’UTR are transported to protrusions by the by Kif1c motor.**

**A**- Micrograph of a fixed NIH/3T3 cell expressing β24bs/Net1 reporter mRNA, MCP-GFP (green), KIF1C-ST_x24_ protein and scFv-mScarletI (red). Single molecules of β24bs/Net1 mRNAs are visible in green, while single molecules of KIF1C-ST_x24_ protein are red. The numbered white boxes are magnified in B. Blue: DNA stained with DAPI. Scale bar is 5 microns.

**B**- Insets represent magnifications of the boxed areas from the image shown in A. Left: MCP-GFP signals labelling β24bs/Net1 mRNAs; middle: scFv-mScarletI labelling KIF1C-ST_x24_ protein; right: merge with mRNAs in green and KIF1C-ST_x24_ in red. Black and white arrowheads indicate colocalization of single molecules of β24bs/Net1 mRNA and KIF1C-ST_x24_. Scale bars are 5 microns.

**C**- Snapshot of a live NIH/3T3 cell expressing β24bs/Net1 mRNA, MCP-GFP (green), KIF1C-ST_x24_ protein and scFv-mScarletI (red). Snapshot is extracted from Movie S10. The white arrowhead indicates a co-transport event of a single molecule of β24bs/Net1 mRNA (green) with a KIF1C-ST_x24_ protein (Red). The boxed area is zoomed in panels D and E. Scale bar is 5 microns.

**D**- Magnification of the boxed area in panel C, highlighting a co-transport event. Top: KIF1C-ST_x24_; Middle: β24bs/Net1 mRNA; bottom: merged panel with the β24bs/Net1 mRNA in green and KIF1C-ST_x24_ in red. Scale bar is 1 micron.

**E**-Kymograph extracted from Movie S10, showing the trajectory of a single molecule of β24bs/Net1 mRNA (left panel), KIF1C-ST_x24_ (middle panel) and the merge (right panel) in NIH/3T3 cells. Total time of the kymograph is 12.9 seconds and corresponds to the event shown in panels C and D.

**Supplementary spreadsheets:**

**Table S1:** Excel file showing the KIF1C-GFP IP microarray data.

**Table S2:** Human smFISH probe sequences and list of probes used for smFISH localization screen in HeLa cells using 18 of the mRNAs most enriched in the KIF1C IP.

**Table S3:** File presenting the source data for the graph presented in Figure S1E. APC-dependence was specified based on reduced protrusion enrichment upon APC knockdown (described in Wang et al, 2017). For each RNA, the fold-change of protrusion enrichment and significance values upon APC knockdown from the Wang et al study are also indicated.

**Table S4:** File presenting the source data for the graphs presented in Figure 6G-H.

**Table S5:** GO term enrichment for the 200 mRNAs most enriched in the KIF1C IP. Analysis was done with http://pantherdb.org/tools/compareToRefList.jsp. The terms highlighted in yellow could relate to the function of KIF1C in cell migration.

**Movie legends:**

**Movie S1:** A live NIH/3T3 cell expressing β24bs/Net1 reporter mRNA and MCP-GFP (green) imaged at 6.67 frames per second for 1 minute, prior to addition of nocodazole. A snapshot of this movie is shown in Figure 3B. Left panel presents the raw signal. Right panel presents the raw signal with overlaid accumulated tracks of individual RNA spots. Only tracks lasting longer than 2.5 seconds are shown. Warmer colors indicate tracks of higher linearity. Scale bar is 5 microns.

**Movie S2:** A live NIH/3T3 cell (same as shown in Movie S1) expressing β24bs/Net1 reporter mRNA and MCP-GFP (green) imaged at 6.67 frames per second for 1 minute, 15 minutes after addition of nocodazole. A snapshot of this movie is shown in Figure 3B. Left panel presents the raw signal. Right panel presents the raw signal with overlaid accumulated tracks of individual RNA spots. Only tracks lasting longer than 2.5 seconds are shown. Warmer colors indicate tracks of higher linearity. Scale bar is 5 microns.

**Movie S3:** A live NIH/3T3 cell expressing β24bs/Net1 reporter mRNA and MCP-GFP (green) imaged at 6.67 frames per second for 1 minute, prior to addition of cytochalasin D. A snapshot of this movie is shown in Figure 3B. Left panel presents the raw signal. Right panel presents the raw signal with overlaid accumulated tracks of individual RNA spots. Only tracks lasting longer than 2.5 seconds are shown. Warmer colors indicate tracks of higher linearity. Scale bar is 5 microns.

**Movie S4:** A live NIH/3T3 cell (same as shown in Movie S3) expressing β24bs/Net1 reporter mRNA and MCP-GFP (green) imaged at 6.67 frames per second for 1 minute, 15 minutes after addition of cytochalasin D. A snapshot of this movie is shown in Figure 3B. Left panel presents the raw signal. Right panel presents the raw signal with overlaid accumulated tracks of individual RNA spots. Only tracks lasting longer than 2.5 seconds are shown. Warmer colors indicate tracks of higher linearity. Scale bar is 5 microns.

**Movie S5:** A live NIH/3T3 cell expressing β24bs/Net1 reporter mRNA and MCP-GFP (green), transfected with control siRNAs, imaged at 6.67 frames per second for 1 minute. A snapshot of this movie is shown in Figure 4A. Left panel presents the raw signal. Right panel presents the raw signal with overlaid accumulated tracks of individual RNA spots. Only tracks lasting longer than 2.5 seconds are shown. Warmer colors indicate tracks of higher linearity. Scale bar is 5 microns.

**Movie S6:** A live NIH/3T3 cell expressing β24bs/Net1 reporter mRNA and MCP-GFP (green), transfected with KIF1C siRNAs, imaged at 6.67 frames per second for 1 minute. A snapshot of this movie is shown in Figure 4A. Left panel presents the raw signal. Right panel presents the raw signal with overlaid accumulated tracks of individual RNA spots. Only tracks lasting longer than 2.5 seconds are shown. Warmer colors indicate tracks of higher linearity. Scale bar is 5 microns.

**Movie S7:** A live NIH/3T3 cell expressing β24bs/Net1 reporter mRNA and MCP-GFP (green), transfected with KIF5B siRNAs, imaged at 6.67 frames per second for 1 minute. A snapshot of this movie is shown in Figure 4A. Left panel presents the raw signal. Right panel presents the raw signal with overlaid accumulated tracks of individual RNA spots. Only tracks lasting longer than 2.5 seconds are shown. Warmer colors indicate tracks of higher linearity. Scale bar is 5 microns.

**Movie S8:** A live NIH/3T3 cell expressing β24bs/Net1 reporter mRNA and MCP-GFP (green), transfected with KIF3A siRNAs, imaged at 6.67 frames per second for 1 minute. A snapshot of this movie is shown in Figure 4A. Left panel presents the raw signal. Right panel presents the raw signal with overlaid accumulated tracks of individual RNA spots. Only tracks lasting longer than 2.5 seconds are shown. Warmer colors indicate tracks of higher linearity. Scale bar is 5 microns.

**Movie S9:** A live NIH/3T3 cell expressing β24bs/Net1 reporter mRNA, MCP-GFP (green), KIF1C-ST_x24_ protein and scFv-mScarletI (red) was imaged at 7.36 frames per second during 6.45 seconds. The movie follows a co-directed transport event highlighted by the white arrowhead in Figure 6C-E. Scale bar is 5 microns. Top left insets are magnifications of the yellow boxed area showing separately each fluorescent channel. Scale bar is 1 micron.

**Movie S10:** A live NIH/3T3 cell expressing β24bs/Net1 reporter mRNA, MCP-GFP (green), KIF1C-ST_x24_ protein and scFv-mScarletI (red) was imaged at 7.36 frames per second during 12.9 seconds. The movie follows a co-directed transport event highlighted by the white arrowhead in Figure S4C-E. Scale bar is 5 microns. Top left insets are magnifications of the yellow boxed area showing separately each fluorescent channel. Scale bar is 1 micron.
